# Use of low-dose rituximab to treat pemphigus^☆^

**DOI:** 10.1016/j.abd.2023.07.019

**Published:** 2024-06-15

**Authors:** Sandra M.B. Durães, Nathália R. Santos, Clarissa N. Batzner, Fernando G.M. Cerqueira

**Affiliations:** a Department of Clinical Medicine, Hospital Universitário Antônio Pedro, Universidade Federal Fluminense, Niterói, RJ, Brazil; b Department of Clinical Medicine, Universidade Federal Fluminense, Niterói, RJ, Brazil

Dear Editor,

Pemphigus is a rare, chronic and relapsing immunobullous disease mediated by anti-desmoglein antibodies that is associated with significant morbidity and mortality.^1^ Currently recommended first-line treatment includes high doses of systemic corticosteroids combined with a corticosteroid-sparing drug ‒ usually azathioprine or mycophenolate mofetil, but also methotrexate, chloroquine or sulfone.^1^ As long-term immunosuppression is needed for adequate disease control, this therapy is linked to serious adverse effects and complications.^1^, ^2^

Rituximab (RTX) is a chimeric anti-CD20 monoclonal antibody that induces B lymphocyte depletion in vivo.^1^ This drug is released by the Brazilian Regulatory Agency to be used in non-Hodgkin's lymphoma, rheumatoid arthritis, chronic lymphoid leukemia, polyangiitis granulomatosis and microscopic polyangiitis. However, it has been used as a treatment of refractory pemphigus, although experience with its use as a first-line corticosteroid-sparing drug is limited.^2^

Recent studies have reported RTX to be effective in refractory Pemphigus Vulgaris (PV) or Pemphigus Foliaceus (PF) as a low-dose protocol. A single course of two infusions of 500 mg RTX with an interval of 2 weeks led to complete remission of the disease in most part of the cases.^3^, ^4^ It represents less than half of the dose used for rheumatologic diseases and also a lower cost of the therapy.^4^ Patients who relapsed were treated with additional cycles of low-dose RTX, resulting in subsequent disease control.^3^, ^4^

We present five patients with pemphigus: three with PV and two with PF. They were all initially treated with the first-line therapy, but due to presenting a refractory condition or aggressive evolution, underwent a protocol with RTX associated or not with adjuvant therapy. Patients’ characteristics, comorbidities, and prior therapy are described in Table 1. This low-dose regimen consisted of 4 infusions of RTX500 mg, administered monthly. However, Patient 2 interrupted the cycle after the second dose due to bureaucratic reasons and, as remission was not achieved, a new cycle was proposed the following year. Remission of the disease means a marked reduction in the severity of its signs and symptoms or its temporary disappearance. Patient 5 developed a pulmonary infection after the second dose, interrupting the treatment. All patients are in follow-up to date. Time since the first infusion, remission range, relapse, and adverse effects are detailed in Table 2.

**Table 1:** Pemphigus patients characteristics treated with low-dose rituximab.

Patient	Gender	Age	Comorbidity	Diagnose	Evolution time (months)	Prior therapy
1	Male	63	Dyslipidemia	PV	84	CS, AZA
2	Female	64	Hypertension, diabetes, dyslipidemia, hypothyroidism	PF	29	CS, CQ, AZA
3	Female	25	‒	PF	37	CS, DDS, AZA
4	Male	46	‒	PV	12	CS, MMF, AZA
5	Male	49	Previous pulmonary tuberculosis	PV	3	CS, AZA

PV, Pemphigus vulgaris; PF, Pemphigus Foliaceus; CS, Systemic Corticosteroids; AZA, Azathioprine; CQ, Chloroquine; DDS, Sulfone; MMF, Mycophenolate Mofetil.

**Table 2:** Outcomes of pemphigus patients treated with low-dose rituximab.

Patient	Date of first infusion	End of the protocol	Remission reach (doses/weeks)	Doses received/weeks in protocol	Disease-free time (months)	Relapse (months)	Adverse effects	Mainteined drugs
1	December/2012	August/2013	4/4	2/36	117	38	‒	Topical corticosteroids
2	May/2014	August/2014	3/66	3/17	99	42	‒	Topical corticosteroids
3	January/2017	April/2017	4/4	4/15	69	‒	‒	‒
4	November/2017	February/2018	4/5	4/16	58	‒	‒	‒
5	February/2018	February/2018	2/8	2/5	57	‒	Pneumonia	‒

As can be seen from these tables, patients achieved disease remission very quickly after the end of the RTX cycle. Only one patient had an adverse effect ‒ mild pulmonary infection, managed outpatiently. We can also observe the low incidence of relapse and prolonged time in remission. Patient 2, who initially received only 2 doses of RTX, went into remission after 4 weeks of the first infusion and remained without new lesions for 5 months. Fourteen months after the first dose, this patient received another cycle of RTX after which achieved remission that lasted 42 months. Currently, there are few lesions that are managed only with topical medications. Patient 1 presented a mild relapse of the disease after 38 months, being treated only with topical corticosteroids.

As pemphigus is a rare disease and RTX is an expensive therapy, there are only a few case series that demonstrate efficacy and few adverse effects, and there is still no evidence regarding the ideal dose and maintenance protocols. Two studies on low-dose RTX in pemphigus used 2 infusions of 500 mg 2 weeks apart. The first reported 15 patients with 6 relapses and the second followed 9 patients with 3 relapses who required retreatment.^3^, ^4^ In a recent study, eight patients received two 200 mg infusions of RTX 14 days apart, which can be considered an ultra-low dose of RTX.^5^

Yet some observations suggest that the early introduction of RTX is even more beneficial. A prospective multicenter study with 90 patients demonstrated that RTX associated with low-dose corticosteroids as a first-line regimen in pemphigus was effective in achieving a faster and longer-lasting remission than in patients who started treatment with standard medication alone.^2^ However, these results could not be observed in our case series, both because of the small sample size and because the protocol could not be followed in all 5 patients. In our small case series, RTX used as low-dose therapy for pemphigus, associated or not with systemic corticosteroids and other immunosuppressants, ensured a fast and lasting remission range, saving patients from potential risks of adverse effects of the first-line treatment. Further studies are needed to determine the best dose, the association with adjuvant therapy, and the risks and costs of this treatment, which has already proved to be advantageous for patients with pemphigus.

## Financial support

None declared.

## Authors’ contributions

Sandra M.B. Durães: Study concepts and design; writing of the manuscript; data interpretation; effective participation in the research guidance studies; intellectual participation in the propaedeutic and/ or therapeutic conduct of studied cases; literature review and final approval of the final version.

Nathália R. Santos: Study concepts and design; data analysis; writing of the manuscript; data interpretation; effective participation in the research guidance studies; literature review and final approval of the final version.

Clarissa N. Batzner: Study concepts and design; data analysis; writing of the manuscript; data interpretation; effective participation in the research guidance studies; literature review and final approval of the final version.

Fernando G.M. Cerqueira: Study concepts and design; data analysis; writing of the manuscript; data interpretation; effective participation in the research guidance studies and literature review.

## Conflicts of interest

None declared.

## References

[1] Frampton J.E. (2020). Rituximab: a review in pemphigus vulgaris. Am J Clin Dermatol.

[2] Joly P., Maho-Vaillant M., Prost-Squarcioni C., Hebert V., Houivet E., Calbo S. (2017). First-line rituximab combined with short-term prednisone versus prednisone alone for the treatment of pemphigus (Ritux 3): a prospective, multicentre, parallel-group, open-label randomised trial. Lancet.

[3] Zhang J., Huang X., Zhang Z., Zhao P., Chen W., Liang Y. (2023). Clinical observation of different doses of rituximab for the treatment of severe pemphigus: a single-center prospective cohort study. J Am Acad Dermatol.

[4] Singh N., Handa S., Mahajan R., Sachdeva N., De D. (2022). Comparison of the efficacy and cost-effectiveness of an immunologically targeted low-dose rituximab protocol with the conventional rheumatoid arthritis protocol in severe pemphigus. Clin Exp Dermatol.

[5] Simpson K., Low Z.M., Yap T., Kern J.S., Scardamaglia L. (2022). Ultralow-dose rituximab in pemphigus: a single-centre experience. Br J Dermatol.

